# Supplementary material for: Translocated populations are genetically similar to natural populations and populations resulting from natural colonizations
Source: PLoS One. 2026 Jan 8;21(1):e0340580. doi: 10.1371/journal.pone.0340580 (PMC12782421; doi:10.1371/journal.pone.0340580)
Supplement: S1 Table — Repeat type is based on genomic DNA sequence analyzed on an Illumina MiSeq platform. Size bp (base pairs) is based on the fragment analysis of 15 individuals on an ABI373. (DOCX) [file pone.0340580.s015.docx]

**Table S1. New microsatellite DNA markers of *Alytes obstetricans* developed by ecogenics GmbH.** Repeat type is based on genomic DNA sequence analyzed on an Illumina MiSeq platform. Size bp (base pairs) is based on the fragment analysis of 15 individuals on an ABI373.

| **Locus** | **Primer sequences 5’ - 3’** | | **Repeat type** | **Size bp** | **No. of alleles** |
| --- | --- | --- | --- | --- | --- |
| Alyobs_01107 | F | GTCTCCCCACTCTACCATGC | (AGAT) 19 | 133 - 145 | 4 |
|  | R | AGTTTAGCATAAAAAGGCCCAC |  |  |  |
| Alyobs_04782 | F | TGTCCCTATCACAAGAACCAAG | (TCTA) 22 | 187 - 223 | 8 |
|  | R | ACAATACAATGTTGCAATCTGGAC |  |  |  |
| Alyobs_06184 | F | TGTAGCAATCCTAGTGGGTC | (TCTA) 10 | 132 - 140 | 3 |
|  | R | ACCTGGCAACTCATTGTCTC |  |  |  |
| Alyobs_08127 | F | TGAGGATCAACAGCCCTACAC | (ATCT) 15 | 177 - 196 | 6 |
|  | R | CATCCTGACCAGGCATACAC |  |  |  |
