# Supplementary material for: Translocated populations are genetically similar to natural populations and populations resulting from natural colonizations
Source: PLoS One. 2026 Jan 8;21(1):e0340580. doi: 10.1371/journal.pone.0340580 (PMC12782421; doi:10.1371/journal.pone.0340580)
Supplement: S2 Table — This table shows eight simulated scenarios for dispersal frequency, population size, and dispersal type. (DOCX) [file pone.0340580.s016.docx]

**Table S2. Details for the simulations made in quantiNemo (v2.0.0) for Emmental and Lucerne populations.** This table shows eight simulated scenarios for dispersal frequency, population size, and dispersal type.

| **Scenario** | **Emmental (natural vs colonized populations)** | **Lucerne (natural vs translocated populations)** |
| --- | --- | --- |
| 1 | Dispersal only happens at the first generation from natural to colonized populations, 5 dispersers and population size limit of 100 individuals. | Constant dispersal among all populations, 5 dispersers and population size limit of  500 individuals. |
| 2 | Constant dispersal from natural to colonized populations, 5 dispersers and  population size limit of 500 individuals. | Constant dispersal among all populations, 5 dispersers and population size limit of  100 individuals. |
| 3 | Constant dispersal from natural to colonized populations, 5 dispersers and  population size limit of 100 individuals. | Dispersal only happens at the first generation among all populations, 5  dispersers and population size limit of 500 individuals. |
| 4 | BAYESSAss dispersal rates only happen at the first generation  among all populations and population size limit of 500 individuals. | Dispersal only happens at the first generation among all populations, 5  dispersers and population size limit of 100 individuals. |
| 5 | BAYESSAss dispersal rates only happen at the first generation  among all populations and population size limit of 100  individuals. | BAYESSAss dispersal rates only happen at the first generation among all  populations and population size limit of 500 individuals. |
| 6 | Constant BAYESSAss dispersal rates among all populations and population  size limit of 500 individuals. | BAYESSAss dispersal rates only happens at the first generation among all  populations and population size limit of 100 individuals. |
| 7 | Constant BAYESSAss dispersal rates among all populations and population  size limit of 100 individuals. | Constant BAYESSAss dispersal values among all populations and population size  limit of 500 individuals. |
| 8 | Dispersal only happens at the first generation from natural to colonized populations, 5 dispersers and population size limit of 500 individuals. | Constant BAYESSAss dispersal values among all populations and population size  limit of 100 individuals. |
| Dispersal frequency | First generation or constant (dispersal in all years) | First generation or constant (dispersal in all years) |
| Population size | 100 or 500 | 100 or 500 |
| Dispersal type | 5 dispersers (from natural to colonized populations) and BAYESSAss dispersal rates (among all populations) | 5 dispersers (among all populations) and BAYESSAss dispersal rates (among all populations) |
