# Supplementary material for: Translocated populations are genetically similar to natural populations and populations resulting from natural colonizations
Source: PLoS One. 2026 Jan 8;21(1):e0340580. doi: 10.1371/journal.pone.0340580 (PMC12782421; doi:10.1371/journal.pone.0340580)
Supplement: S3 Table — A) Shows the p-values of the HWE exact test, Monte Carlo permutations of alleles. Significant deviation from HWE is shown by *(P < 0.05). B) Shows the estimates of null allele frequencies. None of the loci showed consistent deviation from Hardy-Weinberg equilibrium, and null allele frequencies were negligible. (DOCX) [file pone.0340580.s017.docx]

**Table S3. Estimated deviation from Hardy-Weinberg equilibrium (HWE) and null allele frequencies per population and for the 16 microsatellite loci.** A) Shows the p-values of the HWE exact test, Monte Carlo permutations of alleles. Significant deviation from HWE is shown by *(P < 0.05). B) Shows the estimates of null allele frequencies. None of the loci showed consistent deviation from Hardy-Weinberg equilibrium, and null allele frequencies were negligible.

| **A)** | **Region** | **Population/Loci** | **Alyobs_28** | **Alyobs_3** | **Alyobs_4** | **Alyobs_7** | **Alyobs_16** | **Alyobs_8** | **Alyobs_17** | **Alyobs_19** | **Alyobs_20** | **Alyobs_23** | **Alyobs_24** | **Alyobs_25** | **Alyobs_01107** | **Alyobs_04782** | **Alyobs_06184** | **Alyobs_08127** |
| --- | --- | --- | --- | --- | --- | --- | --- | --- | --- | --- | --- | --- | --- | --- | --- | --- | --- | --- |
|  | Emmental | ARM | 1.000 | 1.000 | 1.000 | 0.624 | 1.000 | 1.000 | 1.000 | 1.000 | 1.000 | 0.631 | 1.000 | 1.000 | 1.000 | 1.000 | 1.000 | 0.497 |
|  |  | CHNM | 1.000 | 0.536 | 1.000 | 0.707 | 1.000 | 0.445 | 1.000 | 1.000 | 1.000 | 0.344 | 1.000 | 1.000 | 1.000 | 0.484 | 1.000 | 1.000 |
|  |  | DBM | 1.000 | 0.495 | 1.000 | 1.000 | 1.000 | 1.000 | 1.000 | 1.000 | 1.000 | 0.511 | 1.000 | 1.000 | 0.215 | 1.000 | 1.000 | 1.000 |
|  |  | EGSM | 1.000 | 0.203 | 1.000 | 1.000 | 1.000 | 0.601 | 0.652 | 1.000 | 1.000 | 1.000 | 0.350 | 1.000 | 1.000 | 0.462 | 1.000 | 0.351 |
|  |  | FEM | 1.000 | 0.718 | 1.000 | 1.000 | 0.616 | 0.313 | 0.589 | 1.000 | 1.000 | 1.000 | 0.515 | 1.000 | 0.526 | 0.646 | 1.000 | 0.515 |
|  |  | HNM | 1.000 | 0.740 | 1.000 | 0.871 | 0.330 | 0.010* | 0.645 | 1.000 | 1.000 | 1.000 | 0.671 | 1.000 | 1.000 | 0.079 | 1.000 | 0.044* |
|  |  | HOM | 1.000 | 0.638 | 1.000 | 1.000 | 0.026* | 0.221 | 0.545 | 1.000 | 1.000 | 0.366 | 1.000 | 1.000 | 0.033* | 0.468 | 1.000 | 0.417 |
|  |  | SBM | 1.000 | 1.000 | 1.000 | 1.000 | 1.000 | 1.000 | 1.000 | 1.000 | 1.000 | 0.502 | 1.000 | 1.000 | 1.000 | 0.324 | 1.000 | 0.539 |
|  |  | TFM | 1.000 | 1.000 | 1.000 | 0.045* | 1.000 | 0.003* | 1.000 | 1.000 | 1.000 | 0.307 | 1.000 | 1.000 | 0.084 | 0.161 | 1.000 | 1.000 |
|  |  | SO2M | 1.000 | 1.000 | 1.000 | 1.000 | 1.000 | 0.755 | 1.000 | 1.000 | 1.000 | 1.000 | 1.000 | 1.000 | 1.000 | 1.000 | 1.000 | 0.144 |
|  |  | SO3M | 1.000 | 0.159 | 1.000 | 1.000 | 0.129 | 1.000 | 1.000 | 1.000 | 1.000 | 0.380 | 1.000 | 1.000 | 1.000 | 0.294 | 1.000 | 0.252 |
|  |  | SO7M | 1.000 | 1.000 | 1.000 | 0.549 | 0.663 | 0.578 | 1.000 | 1.000 | 1.000 | 1.000 | 1.000 | 1.000 | 1.000 | 1.000 | 1.000 | 0.630 |
|  |  | S11M | 1.000 | 0.089 | 1.000 | 1.000 | 1.000 | 0.638 | 0.106 | 1.000 | 1.000 | 0.314 | 1.000 | 1.000 | 1.000 | 0.067 | 1.000 | 1.000 |
|  |  | S12M | 1.000 | 0.309 | 1.000 | 1.000 | 0.021* | 0.163 | 1.000 | 1.000 | 1.000 | 0.389 | 0.278 | 1.000 | 1.000 | 1.000 | 1.000 | 0.142 |
|  |  | S13M | 1.000 | 0.132 | 1.000 | 1.000 | 1.000 | 0.194 | 1.000 | 1.000 | 1.000 | 1.000 | 0.336 | 1.000 | 0.135 | 1.000 | 1.000 | 0.010* |
|  |  | S15M | 1.000 | 1.000 | 1.000 | 0.717 | 1.000 | 0.375 | 0.048* | 1.000 | 1.000 | 0.289 | 0.356 | 1.000 | 0.365 | 0.887 | 1.000 | 0.630 |
|  |  | S18M | 1.000 | 1.000 | 1.000 | 0.510 | 1.000 | 0.765 | 1.000 | 1.000 | 1.000 | 0.581 | 1.000 | 1.000 | 1.000 | 0.727 | 1.000 | 1.000 |
|  |  | S21M | 1.000 | 1.000 | 1.000 | 0.298 | 1.000 | 0.828 | 1.000 | 1.000 | 1.000 | 0.519 | 1.000 | 1.000 | 0.649 | 0.504 | 1.000 | 0.282 |
|  |  | S22M | 1.000 | 0.171 | 1.000 | 1.000 | 1.000 | 0.346 | 1.000 | 1.000 | 1.000 | 1.000 | 1.000 | 1.000 | 1.000 | 1.000 | 1.000 | 0.197 |
|  |  | S5BM | 1.000 | 0.600 | 1.000 | 0.656 | 1.000 | 0.295 | 1.000 | 1.000 | 1.000 | 1.000 | 1.000 | 1.000 | 0.230 | 0.137 | 1.000 | 1.000 |
|  |  | S6CM | 1.000 | 0.732 | 1.000 | 0.656 | 1.000 | 0.531 | 1.000 | 1.000 | 1.000 | 0.286 | 0.427 | 1.000 | 0.604 | 0.144 | 1.000 | 0.071 |
|  | Lucerne | KAP | 1.000 | 0.795 | 1.000 | 0.452 | 1.000 | 0.477 | 1.000 | 1.000 | 1.000 | 1.000 | 0.248 | 1.000 | 0.173 | 0.815 | 1.000 | 0.314 |
|  |  | GEI | 1.000 | 1.000 | 1.000 | 0.363 | 0.579 | 0.458 | 1.000 | 1.000 | 1.000 | 1.000 | 1.000 | 1.000 | 0.624 | 0.007* | 1.000 | 0.728 |
|  |  | OTT | 0.604 | 0.650 | 1.000 | 0.660 | 1.000 | 1.000 | 1.000 | 1.000 | 1.000 | 1.000 | 1.000 | 1.000 | 0.648 | 0.292 | 1.000 | 1.000 |
|  |  | CHR | 0.251 | 0.678 | 1.000 | 0.518 | 1.000 | 0.243 | 1.000 | 1.000 | 1.000 | 0.499 | 1.000 | 1.000 | 0.560 | 0.554 | 1.000 | 1.000 |
|  |  | ERS | 1.000 | 0.142 | 1.000 | 0.569 | 0.124 | 0.369 | 0.401 | 1.000 | 1.000 | 0.714 | 1.000 | 1.000 | 0.507 | 0.262 | 1.000 | 1.000 |
|  |  | SON | 1.000 | 0.136 | 1.000 | 0.428 | 0.078 | 0.448 | 0.024* | 1.000 | 1.000 | 0.199 | 0.287 | 1.000 | 0.414 | 0.058 | 1.000 | 1.000 |
|  |  | EHR | 1.000 | 0.031* | 1.000 | 0.510 | 0.733 | 0.890 | 1.000 | 1.000 | 1.000 | 1.000 | 0.554 | 1.000 | 0.748 | 0.157 | 1.000 | 1.000 |
|  |  | CHA | 1.000 | 0.104 | 1.000 | 1.000 | 1.000 | 0.186 | 1.000 | 1.000 | 1.000 | 0.392 | 0.540 | 1.000 | 1.000 | 0.864 | 1.000 | 0.745 |
|  |  | HER | 1.000 | 0.335 | 1.000 | 0.285 | 0.911 | 1.000 | 1.000 | 1.000 | 1.000 | 1.000 | 1.000 | 1.000 | 1.000 | 0.852 | 1.000 | 1.000 |
|  |  | LAT | 1.000 | 0.829 | 1.000 | 0.773 | 0.004* | 0.334 | 1.000 | 1.000 | 1.000 | 1.000 | 0.759 | 1.000 | 1.000 | 0.638 | 1.000 | 0.033* |
|  |  | SSS | 0.705 | 0.120 | 1.000 | 0.236 | 0.714 | 0.419 | 1.000 | 1.000 | 1.000 | 1.000 | 0.369 | 1.000 | 0.414 | 1.000 | 1.000 | 0.143 |
|  |  | STA | 1.000 | 0.727 | 1.000 | 1.000 | 1.000 | 0.852 | 1.000 | 1.000 | 1.000 | 1.000 | 0.514 | 1.000 | 0.289 | 0.640 | 1.000 | 0.887 |
| **B)** | **Region** | **Population/Loci** | **Alyobs_28** | **Alyobs_3** | **Alyobs_4** | **Alyobs_7** | **Alyobs_16** | **Alyobs_8** | **Alyobs_17** | **Alyobs_19** | **Alyobs_20** | **Alyobs_23** | **Alyobs_24** | **Alyobs_25** | **Alyobs_01107** | **Alyobs_04782** | **Alyobs_06184** | **Alyobs_08127** |
|  | Emmental | ARM | ≤0.001 | ≤0.001 | ≤0.001 | ≤0.001 | ≤0.001 | ≤0.001 | ≤0.001 | ≤0.001 | ≤0.001 | ≤0.001 | ≤0.001 | ≤0.001 | ≤0.001 | ≤0.001 | ≤0.001 | ≤0.001 |
|  |  | CHNM | ≤0.001 | 0.079 | ≤0.001 | ≤0.001 | ≤0.001 | ≤0.001 | ≤0.001 | ≤0.001 | ≤0.001 | 0.104 | ≤0.001 | ≤0.001 | ≤0.001 | ≤0.001 | ≤0.001 | ≤0.001 |
|  |  | DBM | ≤0.001 | ≤0.001 | ≤0.001 | ≤0.001 | ≤0.001 | ≤0.001 | ≤0.001 | ≤0.001 | ≤0.001 | ≤0.001 | ≤0.001 | ≤0.001 | ≤0.001 | ≤0.001 | ≤0.001 | ≤0.001 |
|  |  | EGSM | ≤0.001 | 0.019 | ≤0.001 | ≤0.001 | ≤0.001 | 0.047 | 0.032 | ≤0.001 | ≤0.001 | ≤0.001 | 0.025 | ≤0.001 | ≤0.001 | 0.022 | ≤0.001 | ≤0.001 |
|  |  | FEM | ≤0.001 | ≤0.001 | ≤0.001 | ≤0.001 | ≤0.001 | ≤0.001 | 0.042 | ≤0.001 | ≤0.001 | ≤0.001 | 0.032 | ≤0.001 | ≤0.001 | 0.021 | ≤0.001 | ≤0.001 |
|  |  | HNM | ≤0.001 | 0.014 | ≤0.001 | ≤0.001 | ≤0.001 | 0.174 | 0.055 | ≤0.001 | ≤0.001 | ≤0.001 | 0.026 | ≤0.001 | ≤0.001 | ≤0.001 | ≤0.001 | 0.057 |
|  |  | HOM | ≤0.001 | 0.055 | ≤0.001 | ≤0.001 | ≤0.001 | 0.051 | ≤0.001 | ≤0.001 | ≤0.001 | 0.066 | ≤0.001 | ≤0.001 | 0.140 | ≤0.001 | ≤0.001 | ≤0.001 |
|  |  | SBM | ≤0.001 | ≤0.001 | ≤0.001 | ≤0.001 | ≤0.001 | ≤0.001 | ≤0.001 | ≤0.001 | ≤0.001 | ≤0.001 | ≤0.001 | ≤0.001 | ≤0.001 | 0.014 | ≤0.001 | ≤0.001 |
|  |  | TFM | ≤0.001 | ≤0.001 | ≤0.001 | 0.140 | 0.022 | ≤0.001 | ≤0.001 | ≤0.001 | ≤0.001 | ≤0.001 | ≤0.001 | ≤0.001 | 0.135 | ≤0.001 | ≤0.001 | ≤0.001 |
|  |  | SO2M | ≤0.001 | ≤0.001 | ≤0.001 | ≤0.001 | ≤0.001 | ≤0.001 | ≤0.001 | ≤0.001 | ≤0.001 | ≤0.001 | ≤0.001 | ≤0.001 | ≤0.001 | ≤0.001 | ≤0.001 | ≤0.001 |
|  |  | SO3M | ≤0.001 | ≤0.001 | ≤0.001 | ≤0.001 | ≤0.001 | ≤0.001 | ≤0.001 | ≤0.001 | ≤0.001 | 0.067 | ≤0.001 | ≤0.001 | ≤0.001 | ≤0.001 | ≤0.001 | ≤0.001 |
|  |  | SO7M | ≤0.001 | ≤0.001 | ≤0.001 | ≤0.001 | ≤0.001 | ≤0.001 | ≤0.001 | ≤0.001 | ≤0.001 | ≤0.001 | ≤0.001 | ≤0.001 | ≤0.001 | ≤0.001 | ≤0.001 | ≤0.001 |
|  |  | S11M | ≤0.001 | ≤0.001 | ≤0.001 | ≤0.001 | ≤0.001 | ≤0.001 | 0.144 | ≤0.001 | ≤0.001 | ≤0.001 | ≤0.001 | ≤0.001 | ≤0.001 | 0.135 | ≤0.001 | ≤0.001 |
|  |  | S12M | ≤0.001 | 0.043 | ≤0.001 | ≤0.001 | ≤0.001 | ≤0.001 | ≤0.001 | ≤0.001 | ≤0.001 | ≤0.001 | ≤0.001 | ≤0.001 | ≤0.001 | ≤0.001 | ≤0.001 | 0.134 |
|  |  | S13M | ≤0.001 | 0.036 | ≤0.001 | ≤0.001 | ≤0.001 | ≤0.001 | ≤0.001 | ≤0.001 | ≤0.001 | ≤0.001 | ≤0.001 | ≤0.001 | 0.069 | ≤0.001 | ≤0.001 | 0.148 |
|  |  | S15M | ≤0.001 | ≤0.001 | ≤0.001 | ≤0.001 | ≤0.001 | ≤0.001 | 0.007 | ≤0.001 | ≤0.001 | 0.092 | ≤0.001 | ≤0.001 | 0.021 | ≤0.001 | ≤0.001 | ≤0.001 |
|  |  | S18M | ≤0.001 | ≤0.001 | ≤0.001 | 0.082 | ≤0.001 | ≤0.001 | ≤0.001 | ≤0.001 | ≤0.001 | ≤0.001 | ≤0.001 | ≤0.001 | ≤0.001 | ≤0.001 | ≤0.001 | ≤0.001 |
|  |  | S21M | ≤0.001 | ≤0.001 | ≤0.001 | ≤0.001 | ≤0.001 | ≤0.001 | ≤0.001 | ≤0.001 | ≤0.001 | ≤0.001 | ≤0.001 | ≤0.001 | ≤0.001 | ≤0.001 | ≤0.001 | ≤0.001 |
|  |  | S22M | ≤0.001 | ≤0.001 | ≤0.001 | ≤0.001 | ≤0.001 | ≤0.001 | ≤0.001 | ≤0.001 | ≤0.001 | ≤0.001 | ≤0.001 | ≤0.001 | ≤0.001 | ≤0.001 | ≤0.001 | 0.080 |
|  |  | S5BM | ≤0.001 | ≤0.001 | ≤0.001 | ≤0.001 | ≤0.001 | ≤0.001 | ≤0.001 | ≤0.001 | ≤0.001 | ≤0.001 | ≤0.001 | ≤0.001 | ≤0.001 | 0.092 | ≤0.001 | ≤0.001 |
|  |  | S6CM | ≤0.001 | ≤0.001 | ≤0.001 | ≤0.001 | ≤0.001 | ≤0.001 | ≤0.001 | ≤0.001 | ≤0.001 | 0.092 | ≤0.001 | ≤0.001 | 0.037 | ≤0.001 | ≤0.001 | ≤0.001 |
|  | Lucerne | KAP | ≤0.001 | ≤0.001 | ≤0.001 | ≤0.001 | ≤0.001 | 0.006 | ≤0.001 | ≤0.001 | ≤0.001 | ≤0.001 | ≤0.001 | ≤0.001 | 0.063 | ≤0.001 | ≤0.001 | 0.052 |
|  |  | GEI | ≤0.001 | ≤0.001 | ≤0.001 | 0.079 | 0.044 | 0.051 | ≤0.001 | ≤0.001 | ≤0.001 | ≤0.001 | ≤0.001 | ≤0.001 | ≤0.001 | ≤0.001 | ≤0.001 | ≤0.001 |
|  |  | OTT | ≤0.001 | ≤0.001 | ≤0.001 | ≤0.001 | ≤0.001 | ≤0.001 | ≤0.001 | ≤0.001 | ≤0.001 | ≤0.001 | ≤0.001 | ≤0.001 | 0.042 | 0.100 | ≤0.001 | ≤0.001 |
|  |  | CHR | 0.068 | ≤0.001 | ≤0.001 | 0.032 | ≤0.001 | 0.083 | ≤0.001 | ≤0.001 | ≤0.001 | 0.027 | ≤0.001 | ≤0.001 | ≤0.001 | ≤0.001 | ≤0.001 | ≤0.001 |
|  |  | ERS | ≤0.001 | 0.069 | ≤0.001 | ≤0.001 | ≤0.001 | 0.031 | ≤0.001 | ≤0.001 | ≤0.001 | ≤0.001 | ≤0.001 | ≤0.001 | 0.060 | 0.057 | ≤0.001 | ≤0.001 |
|  |  | SON | ≤0.001 | ≤0.001 | ≤0.001 | ≤0.001 | ≤0.001 | ≤0.001 | 0.132 | ≤0.001 | ≤0.001 | ≤0.001 | ≤0.001 | ≤0.001 | 0.031 | ≤0.001 | ≤0.001 | ≤0.001 |
|  |  | EHR | ≤0.001 | 0.034 | ≤0.001 | 0.002 | ≤0.001 | ≤0.001 | ≤0.001 | ≤0.001 | ≤0.001 | ≤0.001 | ≤0.001 | ≤0.001 | ≤0.001 | ≤0.001 | ≤0.001 | ≤0.001 |
|  |  | CHA | ≤0.001 | ≤0.001 | ≤0.001 | ≤0.001 | ≤0.001 | ≤0.001 | ≤0.001 | ≤0.001 | ≤0.001 | ≤0.001 | ≤0.001 | ≤0.001 | ≤0.001 | ≤0.001 | ≤0.001 | ≤0.001 |
|  |  | HER | ≤0.001 | ≤0.001 | ≤0.001 | 0.043 | ≤0.001 | ≤0.001 | ≤0.001 | ≤0.001 | ≤0.001 | ≤0.001 | ≤0.001 | ≤0.001 | ≤0.001 | ≤0.001 | ≤0.001 | ≤0.001 |
|  |  | LAT | ≤0.001 | ≤0.001 | ≤0.001 | ≤0.001 | ≤0.001 | 0.038 | ≤0.001 | ≤0.001 | ≤0.001 | ≤0.001 | ≤0.001 | ≤0.001 | ≤0.001 | ≤0.001 | ≤0.001 | ≤0.001 |
|  |  | SSS | 0.028 | ≤0.001 | ≤0.001 | 0.074 | 0.021 | ≤0.001 | ≤0.001 | ≤0.001 | ≤0.001 | ≤0.001 | 0.069 | ≤0.001 | ≤0.001 | ≤0.001 | ≤0.001 | ≤0.001 |
|  |  | STA | ≤0.001 | ≤0.001 | ≤0.001 | ≤0.001 | ≤0.001 | ≤0.001 | ≤0.001 | ≤0.001 | ≤0.001 | ≤0.001 | ≤0.001 | ≤0.001 | 0.013 | 0.030 | ≤0.001 | ≤0.001 |
