# Supplementary material for: Translocated populations are genetically similar to natural populations and populations resulting from natural colonizations
Source: PLoS One. 2026 Jan 8;21(1):e0340580. doi: 10.1371/journal.pone.0340580 (PMC12782421; doi:10.1371/journal.pone.0340580)
Supplement: S4 Table — The * represents statistically significant results (p < 0.05). L-shaped distributions indicate a stable population with numerous low-frequency alleles, whereas mode-shifted distributions reflect a recent bottleneck, characterized by the loss of rare alleles and a shift toward intermediate allele frequencies. (DOCX) [file pone.0340580.s018.docx]

**Table S4.** **Results on the Bottleneck analysis on natural, colonized and translocated populations from canton Lucerne and Emmental using three different calculation models.** The * represents statistically significant results (*p* < 0.05). L-shaped distributions indicate a stable population with numerous low-frequency alleles, whereas mode-shifted distributions reflect a recent bottleneck, characterized by the loss of rare alleles and a shift toward intermediate allele frequencies.

| **Region** | **Population**  **type** | **Population** | **Sample**  **Size**  **(n)** | **Bottleneck** | | |
| --- | --- | --- | --- | --- | --- | --- |
|  |  |  |  | **Wilcoxon sign rank test** | | **Allele frequency distribution** |
|  |  |  |  | **SMM (Stepwise mutation model)** | **TPM (two-phase model)** |  |
| Emmental | Colonized | ARM | 15 | 0.14844 | 0.05469 | Shifted mode* |
|  |  | CHNM | 8 | 0.285156 | 0.285156 | Shifted mode* |
|  |  | DBM | 10 | 0.031* | 0.031* | Shifted mode* |
|  |  | EGSM | 20 | 0.116211 | 0.080078 | L-shaped |
|  |  | FEM | 17 | 0.001* | 0.001* | Shifted mode* |
|  |  | HNM | 20 | 0.065430 | 0.065430 | L-shaped |
|  |  | HOM | 20 | 0.285156 | 0.248047 | Shifted mode* |
|  |  | SBM | 20 | 0.902344 | 0.902344 | L-shaped |
|  |  | TFM | 15 | 0.037* | 0.037* | Shifted mode* |
|  | Natural | SO2M | 6 | 0.990234 | 0.990234 | L-shaped |
|  |  | S13M | 20 | 0.652344 | 0.577148 | Shifted mode* |
|  |  | S15M | 20 | 0.422852 | 0.347656 | Shifted mode* |
|  |  | S18M | 12 | 0.410156 | 0.410156 | Shifted mode* |
|  |  | S21M | 20 | 0.064453 | 0.024* | L-shaped |
|  |  | S22M | 7 | 0.577148 | 0.577148 | L-shaped |
|  |  | SO3M | 20 | 0.367188 | 0.326172 | Shifted mode* |
|  |  | SO7M | 20 | 0.347656 | 0.347656 | Shifted mode* |
|  |  | S11M | 14 | 0.246094 | 0.187500 | Shifted mode* |
|  |  | S12M | 20 | 0.161133 | 0.116211 | Shifted mode* |
|  |  | S5BM | 20 | 0.371094 | 0.273438 | L-shaped |
|  |  | S6CM | 20 | 0.347656 | 0.384766 | Shifted mode* |
| Lucerne | Translocated | KAP | 30 | 0.993164 | 0.990723 | L-shaped |
|  |  | GEI | 21 | 0.500000 | 0.367188 | L-shaped |
|  |  | OTT | 19 | 0.371094 | 0.125000 | Shifted mode* |
|  |  | CHR | 30 | 0.687500 | 0.460938 | L-shaped |
|  |  | ERS | 30 | 0.137695 | 0.080078 | L-shaped |
|  |  | SON | 30 | 0.284668 | 0.116699 | Shifted mode* |
|  | Natural | EHR | 30 | 0.721680 | 0.539063 | L-shaped |
|  |  | CHA | 30 | 0.751953 | 0.589844 | L-shaped |
|  |  | HER | 30 | 0.926270 | 0.896973 | L-shaped |
|  |  | LAT | 20 | 0.096680 | 0.065430 | Shifted mode* |
|  |  | SSS | 30 | 0.016* | 0.016* | L-shaped |
|  |  | STA | 30 | 0.883789 | 0.838867 | L-shaped |
