# Supplementary material for: Translocated populations are genetically similar to natural populations and populations resulting from natural colonizations
Source: PLoS One. 2026 Jan 8;21(1):e0340580. doi: 10.1371/journal.pone.0340580 (PMC12782421; doi:10.1371/journal.pone.0340580)
Supplement: Attachment — Submitted filename: ResponseToReviewers.docx [file pone.0340580.s020.docx]

Dear Editor,

In this document we explain how we have revised the manuscript in response to the comments made by the editor and the reviewers. Our responses start with RESPONSE >. Line numbers correspond to the files with track changes.

The reviews were very constructive and helped to improve the manuscript.

Best wishes,

on behalf of all authors

------------------------------------------------------------------------------------------------------------------------

PONE-D-25-16547

Translocated populations are genetically similar to natural populations and populations resulting from natural colonizations

PLOS ONE

Dear Dr. Meléndez,

Thank you for submitting your manuscript to PLOS ONE. After careful consideration, we feel that it has merit but does not fully meet PLOS ONE’s publication criteria as it currently stands. Therefore, we invite you to submit a revised version of the manuscript that addresses the points raised during the review process.

RESPONSE > Thank you very much for your comments. We are happy to continue improving this paper. Below, we describe all of the changes we have made in response to your comments and those of the reviewer.

Reviewer 1 made some valuable suggestions, especially regarding the statistical analyses, which should be carefully considered during the revision. Please also make sure that the figures are displayed as intended. In the current version, many of the figures are distorted or incomplete and therefore were not easily reviewed.

RESPONSE > Thank you for the comments. We explain below how we revised the manuscript in response to the comments. Here we would just like to point out that we reran the statistical analysis with Fis as suggested and that we fixed the problem with the figures.

Journal Requirements:

When submitting your revision, we need you to address these additional requirements.

1. Please ensure that your manuscript meets PLOS ONE's style requirements, including those for file naming. The PLOS ONE style templates can be found at

https://journals.plos.org/plosone/s/file?id=wjVg/PLOSOne_formatting_sample_main_body.pdf and

<https://journals.plos.org/plosone/s/file?id=ba62/PLOSOne_formatting_sample_title_authors_affiliations.pdf>.

RESPONSE > We have made the required changes following the suggested templates to fulfil the journal requirements.

2. To comply with PLOS ONE submissions requirements, in your Methods section, please provide additional information regarding the experiments involving animals and ensure you have included details on (1) methods of sacrifice, (2) methods of anesthesia and/or analgesia, and (3) efforts to alleviate suffering.

RESPONSE > We added the request information to the manuscript (from line 97 to 99 and from 132 to 137). Please note that we had an animal welfare permit to conduct the study. In this permit, all animal welfare issues are described and specified.

3. When completing the data availability statement of the submission form, you indicated that you will make your data available on acceptance. We strongly recommend all authors decide on a data sharing plan before acceptance, as the process can be lengthy and hold up publication timelines. Please note that, though access restrictions are acceptable now, your entire data will need to be made freely accessible if your manuscript is accepted for publication. This policy applies to all data except where public deposition would breach compliance with the protocol approved by your research ethics board. If you are unable to adhere to our open data policy, please kindly revise your statement to explain your reasoning and we will seek the editor's input on an exemption. Please be assured that, once you have provided your new statement, the assessment of your exemption will not hold up the peer review process.

RESPONSE > The data used for this study is already available on Zenodo with DOI: https://doi.org/10.5281/zenodo.15052694

4. Thank you for stating the following in the Acknowledgments Section of your manuscript:

“This project was made possible through the funds of the University of Zurich, Department of Evolutionary Biology and Environmental Studies and the Consejo Nacional de Humanidades Ciencias y Tecnologías, Mexico (CONAHCYT).”

We note that you have provided additional information within the Acknowledgements Section that is not currently declared in your Funding Statement. Please note that funding information should not appear in the Acknowledgments section or other areas of your manuscript. We will only publish funding information present in the Funding Statement section of the online submission form.

RESPONSE > We have removed the funding information from the Acknowledgements Section and from the manuscript.

Please remove any funding-related text from the manuscript and let us know how you would like to update your Funding Statement. Currently, your Funding Statement reads as follows:

“This project was made possible through the funds of the University of Zurich, Department of Evolutionary Biology and Environmental Studies and the Consejo Nacional de Humanidades Ciencias y Tecnologías, Mexico (CONAHCYT).”

Please include your amended statements within your cover letter; we will change the online submission form on your behalf.

RESPONSE > The Funding Statement we have provided is correct. We don’t wish to do any amendments.

5. We note that Figure 1 in your submission contain [map/satellite] images which may be copyrighted. All PLOS content is published under the Creative Commons Attribution License (CC BY 4.0), which means that the manuscript, images, and Supporting Information files will be freely available online, and any third party is permitted to access, download, copy, distribute, and use these materials in any way, even commercially, with proper attribution. For these reasons, we cannot publish previously copyrighted maps or satellite images created using proprietary data, such as Google software (Google Maps, Street View, and Earth). For more information, see our copyright guidelines: http://journals.plos.org/plosone/s/licenses-and-copyright.

We require you to either (1) present written permission from the copyright holder to publish these figures specifically under the CC BY 4.0 license, or (2) remove the figures from your submission:

a. You may seek permission from the original copyright holder of Figure 1 to publish the content specifically under the CC BY 4.0 license.

We recommend that you contact the original copyright holder with the Content Permission Form (http://journals.plos.org/plosone/s/file?id=7c09/content-permission-form.pdf) and the following text:

“I request permission for the open-access journal PLOS ONE to publish XXX under the Creative Commons Attribution License (CCAL) CC BY 4.0 (http://creativecommons.org/licenses/by/4.0/). Please be aware that this license allows unrestricted use and distribution, even commercially, by third parties. Please reply and provide explicit written permission to publish XXX under a CC BY license and complete the attached form.”

Please upload the completed Content Permission Form or other proof of granted permissions as an "Other" file with your submission.

In the figure caption of the copyrighted figure, please include the following text: “Reprinted from [ref] under a CC BY license, with permission from [name of publisher], original copyright [original copyright year].”

RESPONSE > We made a new map using data which is all free to use. Specifically, we used map data which only uses publicly available data. The data is available in the R package “rnaturalearth” and “rnaturalearthdata”.

b. If you are unable to obtain permission from the original copyright holder to publish these figures under the CC BY 4.0 license or if the copyright holder’s requirements are incompatible with the CC BY 4.0 license, please either i) remove the figure or ii) supply a replacement figure that complies with the CC BY 4.0 license. Please check copyright information on all replacement figures and update the figure caption with source information. If applicable, please specify in the figure caption text when a figure is similar but not identical to the original image and is therefore for illustrative purposes only.

RESPONSE > We made a new map for which we do not need a permit.

The following resources for replacing copyrighted map figures may be helpful:

USGS National Map Viewer (public domain): http://viewer.nationalmap.gov/viewer/

The Gateway to Astronaut Photography of Earth (public domain): http://eol.jsc.nasa.gov/sseop/clickmap/

Maps at the CIA (public domain): https://www.cia.gov/library/publications/the-world-factbook/index.html and https://www.cia.gov/library/publications/cia-maps-publications/index.html

NASA Earth Observatory (public domain): http://earthobservatory.nasa.gov/

Landsat: http://landsat.visibleearth.nasa.gov/

USGS EROS (Earth Resources Observatory and Science (EROS) Center) (public domain): http://eros.usgs.gov/#

Natural Earth (public domain): http://www.naturalearthdata.com/

RESPONSE > Thank you for the suggested resources. However, we used a different package in R for which we do not need a permit.

Reviewers' comments:

Reviewer's Responses to Questions

**Comments to the Author**

1. Is the manuscript technically sound, and do the data support the conclusions?

The manuscript must describe a technically sound piece of scientific research with data that supports the conclusions. Experiments must have been conducted rigorously, with appropriate controls, replication, and sample sizes. The conclusions must be drawn appropriately based on the data presented.

Reviewer #1: Yes

2. Has the statistical analysis been performed appropriately and rigorously?

Reviewer #1: I Don't Know

3. Have the authors made all data underlying the findings in their manuscript fully available?

The [PLOS Data policy](http://www.plosone.org/static/policies.action#sharing) requires authors to make all data underlying the findings described in their manuscript fully available without restriction, with rare exception (please refer to the Data Availability Statement in the manuscript PDF file). The data should be provided as part of the manuscript or its supporting information, or deposited to a public repository. For example, in addition to summary statistics, the data points behind means, medians and variance measures should be available. If there are restrictions on publicly sharing data—e.g. participant privacy or use of data from a third party—those must be specified.

Reviewer #1: Yes

4. Is the manuscript presented in an intelligible fashion and written in standard English?

PLOS ONE does not copyedit accepted manuscripts, so the language in submitted articles must be clear, correct, and unambiguous. Any typographical or grammatical errors should be corrected at revision, so please note any specific errors here.

Reviewer #1: Yes

5. Review Comments to the Author

Please use the space provided to explain your answers to the questions above. You may also include additional comments for the author, including concerns about dual publication, research ethics, or publication ethics. (Please upload your review as an attachment if it exceeds 20,000 characters)

Reviewer #1: This paper provides a valuable contribution to understanding the genomic patterns of naturally and artificially colonized ponds, and compares them to original source populations. I appreciate the authors' use of existing opportunities to assess the genomic status of an organism of conservation concern, a commendable approach to monitoring these populations. The manuscript employs a range of population genetic tools and statistical approaches to assess and project genetic diversity across population types. Overall, the methods appear thoughtfully selected and generally appropriate for the study's objectives. I offer the following comments and suggestions to help improve clarity and robustness:

RESPONSE > Thank you for your positive assessment of our manuscript.

Introduction:
The statement, “Beatrice Lüscher made sure that no translocations took place in these naturally colonised populations,” is mentioned. However, without additional context regarding who Beatrice Lüscher is outside the author section and how they ensured no translocations, it is difficult to fully understand the significance of this statement.

RESPONSE > We added the information to the manuscript that Beatrice Lüscher is the local amphibian conservation officer.

Sampling:
The study uses a good narrow sampling timeframe, which is especially relevant for a species that reproduces annually. This strengthens the validity of their results.
One point of clarification: I am unsure if the tadpoles sampled survived the sampling process. If the organisms do not survive, it would be helpful to specify this to avoid any potential confusion.
RESPONSE > It is a threatened species. Therefore, there was no lethal sampling. This is now explained in the section “Research permits and ethical consideration”.

Methods:
The laboratory methods used are sound, and bioinformatics techniques for assessing linkage disequilibrium (LD), Hardy-Weinberg equilibrium (HWE), null alleles, and percent missing data appear appropriate and well-executed. For genetic diversity and bottleneck detection, AR and private alleles are strongly associated with sample size. Larger sample sizes typically contain more alleles compared to smaller samples. It seems in your results that this pattern doesn't obscure your results, which I think makes them even more meaningful. The authors use mean Fis to assess whether it significantly differs from zero. However, Fis values are often not normally distributed across microsatellites. Given this, I suggest using the median Fis to summarize the data, as it may offer a more robust estimate, or demonstrate normality.
RESPONSE > We used the median Fis values as suggested by the reviewer to assess whether it significantly differs from zero. Only the result for Lucerne natural populations changed from no significant to significantly different from zero (line 157; from line 259 to 264; line 362; Table S1).

Statistical Analyses:
The use of linear models to compare genetic diversity indicators across regions and population types is valid for continuous response variables. However, I recommend clarifying whether the model assumptions (e.g., normality) were checked, particularly for metrics like Fst and Fis. These metrics may require transformations before fitting a linear model, and this should be addressed in the methods section.

RESPONSE > The model assumption of normality of residuals was checked for each model. For all models, the null hypothesis of normality was not rejected (From line 172 to 175).

On line 160, the authors suggest that the Ne values reaching infinity could be due to sampling error. I encourage the authors to review the literature for other possible explanations of this phenomenon, as it may not be solely a result of sampling error.

RESPONSE > We have added two possible more explanations in accord to our findings (from line 162 to 166).

The use of BOTTLENECK is appropriate for detecting recent bottlenecks, and the authors selected models suited to microsatellite data. However, I recommend reporting the specific significant p-values, rather than just using the asterisk notation, to allow for clearer interpretation. Additionally, given the weight placed on bottlenecks in the discussion, the results section could benefit from more detailed reporting of the bottleneck results, including data from the supplemental section.

RESPONSE > We have added the p-values in Table S4 for the models SMM and TPM. For the allele frequency distribution there is not p-values but frequencies across loci and tends to follow an L-shaped distribution a shifted mode. After a recent bottleneck rare alleles are lost more rapidly than intermediate-frequency alleles. This shifts the allele frequency distribution. This is known as shifted mode. So, we can only report if the frequencies of alleles across loci have a L-shape distribution or a shifted mode (presence of bottleneck). This all is explained in the references we cited in the manuscript with number 69, 70, 71 and 72. We also added in the Result Section (from line 272 to 277) more detail on the bottleneck results.

The use of quantiNemo to simulate genetic diversity loss under different demographic and dispersal scenarios is a strong approach for understanding future genetic diversity trajectories. However, I recommend clarifying whether multiple replicates were simulated for each scenario, as stochasticity plays a significant role in forward-time simulations.

RESPONSE > We have added that we run 100 replicates for each scenario (line 226).


Figures:
I was unable to view Figure 1 properly, and I expect the version in the PDF is distorted compared to what was submitted. However, even considering that, the figure is tricky to interpret. I only see orange dots on the main map, and I wonder if the purple dots are covered. If the final version of the figure does not contain purple dots, this should be addressed. In addition, the inset map could benefit from using the same color-coding as the main map. To make the figure more accessible at a glance, I recommend changing the shape of the dots for different site types, in addition to using lowercase letters for differentiation.

RESPONSE > we created a new map.


Supplementary Information:
I believe Supplemental Table 1 provides important information that would benefit from being moved to the main paper rather than remaining in the supplements. Its content appears central to the study and would improve the overall readability of the paper if included in the main manuscript.

RESPONSE > We moved Table S1 to the main text. It is now Table 1.

Supplemental Table 4 is difficult to read due to formatting issues. Despite attempts to adjust the table in my Word document, I was unable to view all the columns at once. I recommend improving the formatting for better accessibility.

RESPONSE > We changed the format of the table. It is now much easier to read. However, the table is hard to read because it contains a lot of information.


Overall, this paper presents a compelling study on genetic diversity patterns in a conservation context, and the methodology is generally sound. A few clarifications and additions would strengthen the manuscript, particularly in terms of statistical analysis and presentation of results. The authors use of current genetic diversity data and forward simulations is a notable strength, and I believe these findings will make a valuable contribution to the field.

RESPONSE > Thank you for the positive evaluation. We have tried to improve the presentation of the results.

6. PLOS authors have the option to publish the peer review history of their article ([what does this mean?](https://journals.plos.org/plosone/s/editorial-and-peer-review-process#loc-peer-review-history)). If published, this will include your full peer review and any attached files.


If you choose “no”, your identity will remain anonymous but your review may still be made public.


**Do you want your identity to be public for this peer review?** For information about this choice, including consent withdrawal, please see our [Privacy Policy](https://www.plos.org/privacy-policy).

Reviewer #1: No

[NOTE: If reviewer comments were submitted as an attachment file, they will be attached to this email and accessible via the submission site. Please log into your account, locate the manuscript record, and check for the action link "View Attachments". If this link does not appear, there are no attachment files.]

While revising your submission, please upload your figure files to the Preflight Analysis and Conversion Engine (PACE) digital diagnostic tool, <https://pacev2.apexcovantage.com/>. PACE helps ensure that figures meet PLOS requirements. To use PACE, you must first register as a user. Registration is free. Then, login and navigate to the UPLOAD tab, where you will find detailed instructions on how to use the tool. If you encounter any issues or have any questions when using PACE, please email PLOS at [figures@plos.org](mailto:figures@plos.org). Please note that Supporting Information files do not need this step.

*In compliance with data protection regulations, you may request that we remove your personal registration details at any time.* [*(Remove my information/details)*](https://www.editorialmanager.com/pone/login.asp?a=r)*. Please contact the publication office if you have any questions.*
