# Supplementary material for: Translocated populations are genetically similar to natural populations and populations resulting from natural colonizations
Source: PLoS One. 2026 Jan 8;21(1):e0340580. doi: 10.1371/journal.pone.0340580 (PMC12782421; doi:10.1371/journal.pone.0340580)
Supplement: Attachment — Submitted filename: ResponseToReviewers_auresp_2.docx [file pone.0340580.s021.docx]

Dear Editor,

Thank you for giving us another opportunity to revise our manuscript. In this document we explain how we have revised the manuscript in response to the comments made by the editor and the reviewers. Our responses start with RESPONSE >. Line numbers correspond to the files with track changes.

We deleted text from the previous decision letter that is not relevant here.

The reviews were very constructive and helped to improve the manuscript.

Best wishes,

on behalf of all authors

------------------------------------------------------------------------------------------------------------------------

| **Subject:** | PLOS ONE Decision: Revision required [PONE-D-25-16547R1] |
| --- | --- |

PONE-D-25-16547R1

Translocated populations are genetically similar to natural populations and populations resulting from natural colonizations

PLOS ONE

Dear Dr. Meléndez-Cal-y-Mayor,

Thank you for submitting your manuscript to PLOS ONE. After careful consideration, we feel that it has merit but does not fully meet PLOS ONE’s publication criteria as it currently stands. Therefore, we invite you to submit a revised version of the manuscript that addresses the points raised during the review process.

I agree with the new reviewer that the manuscript did greatly improve and that many of the previous comment have been addressed but there are still a few comment that have not been answered thoroughly enough.

RESPONSE > Thank you. Below we explain how we improved the manuscript. In the last revision, we gave the most consideration to those comments that served to improve the answers to our scientific questions.

Please submit your revised manuscript by Dec 06 2025 11:59PM. If you will need more time than this to complete your revisions, please reply to this message or contact the journal office at [plosone@plos.org](mailto:plosone@plos.org). When you're ready to submit your revision, log on to https://www.editorialmanager.com/pone/ and select the 'Submissions Needing Revision' folder to locate your manuscript file.

Please include the following items when submitting your revised manuscript:

- A rebuttal letter that responds to each point raised by the academic editor and reviewer(s). You should upload this letter as a separate file labeled 'Response to Reviewers'.
- A marked-up copy of your manuscript that highlights changes made to the original version. You should upload this as a separate file labeled 'Revised Manuscript with Track Changes'.
- An unmarked version of your revised paper without tracked changes. You should upload this as a separate file labeled 'Manuscript'.

We look forward to receiving your revised manuscript.

Kind regards,

Sven Winter
Academic Editor
PLOS ONE

Journal Requirements:

If the reviewer comments include a recommendation to cite specific previously published works, please review and evaluate these publications to determine whether they are relevant and should be cited. There is no requirement to cite these works unless the editor has indicated otherwise. 

Please review your reference list to ensure that it is complete and correct. If you have cited papers that have been retracted, please include the rationale for doing so in the manuscript text, or remove these references and replace them with relevant current references. Any changes to the reference list should be mentioned in the rebuttal letter that accompanies your revised manuscript. If you need to cite a retracted article, indicate the article’s retracted status in the References list and also include a citation and full reference for the retraction notice.

RESPONSE > We have checked our reference list, and they are complete and correct.

6. Review Comments to the Author

Please use the space provided to explain your answers to the questions above. You may also include additional comments for the author, including concerns about dual publication, research ethics, or publication ethics. (Please upload your review as an attachment if it exceeds 20,000 characters)

Reviewer #2: I have carefully examined the authors’ point-by-point responses to the previous review as well as the revised version of the manuscript. Overall, it is clear that the authors have made a substantial effort to address the earlier concerns, and many of the revisions have improved the clarity, methodological transparency, and overall quality of the paper. In particular, the handling of the statistical comments appears sound: the use of median Fis values was implemented as suggested, model assumptions were tested and reported, and the treatment of bottleneck results has been improved. Ethical aspects related to animal handling have been clarified with commendable precision, and the data availability now meets the journal’s requirements. These are all meaningful improvements.

The manuscript itself presents a well-defined and relevant study that addresses the important question of genetic similarity of translocated, colonized, and natural populations. The research is well framed and generally methodologically solid. The use of multiple analytical approaches and the integration of empirical data with forward simulations are notable strengths that make this study potentially valuable to the conservation genetics community.

RESPONSE > Thank you for the positive assessment of the manuscript and the revision.

However, some of the responses remain rather superficial. This is especially true for the interpretation of infinite Ne values, which is acknowledged but not convincingly discussed,

RESPONSE > We added more specific explanations of the potential reasons why we found infinite Ne values based on previous works and on our data (from line 160 to 165). We explain that we think that infinite Ne are most likely an artefact.

and for the section on bottleneck events, where additional depth and contextualization would strengthen the argument

RESPONSE > We added more additional information to strengthen our argument (from line 372 to 385). We explain why we believe that there were bottlenecks in the recently colonized populations (simply because colonization caused the bottleneck). In one of the “old” natural populations in Lucerne area, we believe it was due to maintenance of the well.

(please also check all references again, some are missing, e.g., l. 180 "residuals were tested for each model using the function jarqueberaTest () and dwtest ()").

RESPONSE > The citation brackets in PlosOne are []. The brackets that the reviewer mentioned as lacking a citation correspond to the functions jarqueberaTest and dwtest (it is R language). These brackets indicate that both are functions, and inside the brackets, the parameters are specified. The functions jarqueberaTest and dwtest do not require a citation because they are included in the fBasics package, which is already cited (lines 176-177).

The adjustments to figures and tables still fall short of making the results fully accessible at a glance. For example, the revised map is likely clearer than the original, but the description suggests that the overall visualization strategy remains basic rather than genuinely improved, and the whole map (still?) appears broken to me. Please check your visualization tool.

RESPONSE > It is a simple map which shows the location of the adjacent study areas and the populations. We used different colours for the two study areas. We believe that the map serves its purpose. We double-checked all files during the submission process to make sure that the maps are displayed correctly.

Moreover, all figures and tables need a proper caption, which is self-explanatory; e.g., table 1 lacks a conclusive caption.

RESPONSE > We have included more details in the captions of all figures and tables.

A general pattern throughout the responses is that several replies address the comments formally, but without fully exploiting the opportunity to clarify or deepen the presentation of the results. For example, the discussion of the infinite Ne values just lists two additional explanations without critically evaluating their likelihood in the specific study context or providing supporting literature.

RESPONSE > We added some additional explanations for Ne and bottlenecks. We don’t think that a further discussion is useful because it would be speculative. Regarding the infinite Ne values, we added additional explanations and supporting literature in the manuscript (from line 160 to 165). This new information explains the possible reasons why the Ne value was infinite for each population.

The response to the bottleneck-related comment adds some details but remains descriptive and lacks an interpretation of how these results inform the study’s main conclusions.

RESPONSE > We added in the manuscript more interpretation on the presence of bottleneck in 3 colonized populations and in a natural population (from line 374 to 385). This connects with the discussion we do on the presence of bottleneck from line 424 to 430. The main conclusion is that the three population types do not differ much despite the bottlenecks. We added some text in the discussion which clarifies this.

Likewise, the comment on improving figure clarity was addressed by creating a new map, but without any clear rationale for how the new visualization strategy better communicates the underlying patterns.

RESPONSE > We believe that the new maps convey that necessary information. Fig 1 shows the two study areas and all the populations while Fig 3 shows that spatial genetic structure. The maps are simple, but all the necessary information is there. There is no additional information in the maps that may distract (e.g. Hill shade).

While the core analyses seem sound and the technical points were largely addressed, the interpretation and communication of the findings could still be more thorough.

RESPONSE > We added additional explanations in the discussion in response to the previous comments and hope that this made the interpretation and discussion of the results more thorough.

In sum, the authors have clearly engaged with the reviewers’ feedback and improved the manuscript in several important respects.

RESPONSE > Thank you.

Yet some areas would benefit from more thoughtful revision, particularly regarding interpretation of key genetic results (biological context) and data presentation. I would therefore recommend minor revisions, with a focus on strengthening the clarity and depth of interpretation as well as improving the accessibility of figures and tables.

RESPONSE > We explain in the replies to the previous comments how we improved the manuscript.

7. PLOS authors have the option to publish the peer review history of their article ([what does this mean?](https://journals.plos.org/plosone/s/editorial-and-peer-review-process#loc-peer-review-history)). If published, this will include your full peer review and any attached files.

If you choose “no”, your identity will remain anonymous but your review may still be made public.

**Do you want your identity to be public for this peer review?** For information about this choice, including consent withdrawal, please see our [Privacy Policy](https://www.plos.org/privacy-policy).

Reviewer #2: No

[NOTE: If reviewer comments were submitted as an attachment file, they will be attached to this email and accessible via the submission site. Please log into your account, locate the manuscript record, and check for the action link "View Attachments". If this link does not appear, there are no attachment files.]

To ensure your figures meet our technical requirements, please review our figure guidelines: https://journals.plos.org/plosone/s/figures

You may also use PLOS’s free figure tool, NAAS, to help you prepare publication quality figures: https://journals.plos.org/plosone/s/figures#loc-tools-for-figure-preparation.

NAAS will assess whether your figures meet our technical requirements by comparing each figure against our figure specifications.

*In compliance with data protection regulations, you may request that we remove your personal registration details at any time.* [*(Remove my information/details)*](https://www.editorialmanager.com/pone/login.asp?a=r)*. Please contact the publication office if you have any questions.*
